# Factors related to sustainable employment of people with acquired brain injury or spinal cord injury: The employer's perspective

**DOI:** 10.3389/fresc.2022.876389

**Published:** 2022-08-02

**Authors:** Barbara Schiffmann, Monika E. Finger, Katarzyna Karcz, Stefan Staubli, Bruno Trezzini

**Affiliations:** ^1^Swiss Paraplegic Research, Nottwil, Switzerland; ^2^Department of Health Sciences and Medicine, University of Lucerne, Lucerne, Switzerland; ^3^Swiss Paraplegic Centre, Nottwil, Switzerland

**Keywords:** sustainable employment, vocational rehabilitation, qualitative study, spinal cord injury (SCI), acquired brain injury (ABI), employer's perspective, disability and work

## Abstract

**Background:**

Of those people with an acquired brain injury (ABI) or spinal cord injury (SCI) who initially successfully returned to paid employment, some exit the workforce before reaching official retirement age. Employers play a central role in ensuring a sustainable work situation for employees with a disability and in preventing such exits. However, the factors and mechanisms involved from the employer's perspective are still poorly understood.

**Purpose:**

The purpose was to determine factors which, from employer's perspective, have a particularly positive or negative influence on sustainable employment of people with ABI or SCI.

**Methods:**

Twenty semi-structured interviews were conducted with employers of people with ABI or SCI and thematically analyzed.

**Results:**

Identified factors could be assigned to four thematic areas for both health conditions: socio-demographic and psychological characteristics of the disabled person, their work performance, the work environment, and other social/environmental conditions. Good disability self-management and proactive communication of needs on the part of the employee are contributing factors to long-term employment from the employer's perspective. Differing expectations and assessments of work performance by employees and employers pose a challenge. Employers feel a responsibility to provide an optimal work environment to allow the employee with a disability to reach his or her full potential. This includes appropriate work tasks, development opportunities, a compassionate work team, flexible work arrangements, providing resources to address specific needs, and an inclusive culture. Employers find the support provided by occupational specialists very helpful, as they often lack the knowledge to design the work environment to meet the person's needs.

**Conclusions:**

Employers emphasize the benefits of professional support during vocational rehabilitation to prepare employers and employees for long-term, sustainable employment. Such support is often lacking when changes and problems occur at a later stage. Therefore, people with a disability should be able to communicate their work-related needs and take charge of their own health so that problems that arise can be addressed as early as possible. Continued awareness of the environment is also beneficial. In addition, the expansion of low-threshold health-specific support services for long-term problems was found to be of great importance for employers in Switzerland.

## Introduction

Spinal Cord Injury (SCI; traumatic or non-traumatic etiology) and acquired brain injury (ABI; traumatic brain and non-traumatic injury such as stroke) are considered to be global health priorities with a global estimated prevalence of 22.5 million cases for SCI and 151 million cases for ABI (head injury and stroke) in 2017 (1). With focus on Switzerland, there are ~6,000 people with a spinal cord injury and 130,000 people with an acquired brain injury (2–4). Both SCI and ABI are neurological conditions that cause the most years of healthy life lost due to disability (YLD) worldwide compared to other conditions (5). The consequences of both these injuries are manifold and often affect the ability to work (6, 7). Vocational rehabilitation measures contribute to the return to work by supporting and accompanying the person with a disability and their work environment on this path. The importance of work participation for the social inclusion of persons with a disability has long been highlighted by disability advocacy groups and is increasingly also recognized in research and policy worldwide (8).

In Switzerland, the employment rate of people with SCI, at around 61%, is significantly lower than that of the population as a whole, at 80% (9). However, it is among the highest in international comparison, where the average employment rate is 38% (10). Nevertheless, long-term data on the employment rate of persons with SCI point to a possible drop-out problem. For example, a longitudinal study found that of 311 people with SCI who were still in paid work in 2012, 37 (12%) were no longer working 5 years later, although they were still of working age (11). Of these 37 participants, 25 were younger than 55, whereas early retirement age in the general population begins at 58 and only 5% retire before age 60 (12). Regular retirement age in Switzerland is 64 for women and 65 for men. In addition, over the 5-year period, 49 persons reduced their workload. This cannot be explained with the unemployment situation in Switzerland being only at 3.1% for the working population for 2015 to 2017 (13). Statistical data on the employment of persons with ABI in Switzerland are not available. Evidence of early withdrawal from work by people with ABI or SCI also comes from patient organizations, such as Fragile Suisse or the Swiss Paraplegic Association. They report, for example, enquiries from affected persons who contact them after losing their job.

Existing scientific evidence shows which factors facilitate or hinder successful vocational reintegration. They also point to the importance of early vocational interventions in an interdisciplinary setting for successful vocational reintegration (14–18). However, there is a lack of in-depth knowledge on what happens after reintegration in the long-term, and what is important for a sustainable work situation (19). Under a sustainable work situation we understand a situation where: “a person–job–workplace match enables a person to stay healthy and satisfied at work over time, with a work performance that meets the expectations of the person and the employer” (20). The lack of knowledge on sustainable employment in the long-term for the Swiss context may partly be due to the fact that in Switzerland, as in most countries, there is no routine professional follow-up after the completion of vocational reintegration. and the determination of eligibility for a partial or full pension, which is mainly decided through the Swiss Disability Insurance (IV) at the end of vocational reintegration. Two scoping reviews on sustaining work after a brain or spinal cord injury identified the few studies that shed light on the period after reintegration in the international context (21, 22). However, none of the studies addresses the employers' perspective.

Employers play an important role in the reintegration of people with a disability and in their long-term employment. There is a growing body of research on the employers' perspectives, attitudes and practices in (not) hiring persons with a disability (23–27). In a recent review, Bonaccio et al. (28) address the various fears of employers when hiring, employing and dismissing a person with a disability and counter these with scientific evidence on the topic of work and disability. However, they do not address disability-specific challenges that may lead to additional insecurities for employees with ABI or SCI and their employers. It is also unclear to what extent their findings apply to Switzerland, as there are no specific laws protecting employees with a disability and the structure of the economy differs from most countries in that 99% of the companies employ fewer than 250 people (29). Thus, in Switzerland, there is a lack of in-depth discussion of the views and perceptions of employers who themselves have experience with employees with ABI or SCI.

The research question of the study is thus as follows: From the employers' perspective, what factors promote or hinder sustainable employment of people with ABI or SCI? In addition, the inclusion of two disability groups makes it possible to identify commonalities and disability-specific differences. Commonalities refer to aspects with potential for generalization to other types of disability. Differences refer to peculiarities of the disability group and its environment that need to be considered.

This study is part of a research project on sustainable employment of people with ABI or SCI in the long-term, in which the topic is approached from different perspectives through triangulation: from the perspective of those affected (30), the perspective of health professionals (31) and the perspective of employers.

## Materials and methods

To answer the research question, qualitative interviews were conducted with employers at their workplace and analyzed using thematic analysis (32, 33).

### Recruitment strategy and sample

For the study we used a purposive sample with a maximum variation (34): employers were sought who employed at least one person with ABI or SCI at the time of the interview or had employed them in the past. In addition, different types of companies (private, public sector, non-profit) and company sizes were to be represented among the employers interviewed. We took care to ensure that at least two companies were always represented within the various criteria, see Table 1. With the maximum variation sample, patterns and aspects that emerge across the different interviews are of particular interest, since they show the core experiences and the central themes regarding the research question (34).

**Table 1:** Description of the sample.

Interview identification number*	**Gender of interviewed employer**	**Company type**	Company size**	**Direct superior?**	**Person with a disability employed by company before injury**	**Number of employees with SCI/ABI**	**Duration of interview (rounded off)**
ABI_01	Female	Foundation	Small	Yes	No	3	40
ABI_02	Male	Private enterprise	Medium	No/HR	Yes	2	40
ABI_03	Male	Public sector	Small	Yes	No	1	45
ABI_04	Female	Private enterprise	Medium	No/HR	Varying	2	50
ABI_05	Male	Private enterprise	Small	No	No	1	35
ABI_06	Female	Public sector	Large	Yes	Yes	1	45
ABI_07	Male	Public sector	Medium	Yes	Yes	1	45
ABI_08	Female	Public sector	Large	Yes	Yes	1	60
ABI_09	Female	Public sector	Large	Yes	No	2	50
ABI_10	Male	Private enterprise	Medium	Yes	Yes	1	95
SCI_01	Male	Public sector	Medium	Yes	No	1	35
SCI_02	Male	Private enterprise	Small	Yes	No	1	30
SCI_03	Male	Private enterprise	Medium	Yes	No	1	55
SCI_04	Male	Private enterprise	Large	No	Yes	1	35
SCI_05	Male	Foundation	Small	Yes	Yes	1	35
SCI_06	Male	Private enterprise	Large	No/HR	Yes	2	50
SCI_07	Female	Private enterprise	Micro	Yes	Varying	2	55
SCI_ABI_01	Male	Foundation	Small	Yes	Varying	2	70
SCI_ABI_02	Male	Private enterprise	Small	Yes	Yes	1	50
SCI_ABI_03	Male	Foundation	Large	No	No	3	60

*ABI, acquired brain injury; SCI, spinal cord injury.

**Company Size: Micro: 1 to 9 employees; small: 10 to 49 employees; medium: 50 to 249 employees; large: 250 and more employees (Swiss Federal Statistical Office, Small and Medium-sized Enterprises, https://www.bfs.admin.ch/bfs/de/home/statistiken/industrie-dienstleistungen/unternehmen-beschaeftigte/wirtschaftsstruktur-unternehmen/kmu.html). Interview identification number: E = employer; A = acquired brain injury; S = spinal cord injury.

We pursued different strategies to recruit employers. For example, people with ABI or SCI who participated in two other sub-studies of the overall research project conducted by the authors were asked if we could contact their employers. In addition, ParaWork, the unit which is responsible for the occupational reintegration of patients at the Swiss Paraplegic Centre, Impulse, an NGO focusing on work integration of people with a disability, and health professionals who participated in another sub-study, provided leads to suitable interview partners who they had supported in the integration of an affected employee. The participating organizations received a flyer to inform potentially interested people about the study. In addition, blog posts and flyers were posted on websites and in the newsletter of Fragile.ch, the newsletter of Compasso (an employers' network) and the online community of the Swiss Paraplegic Society in order to recruit interested employers. Employers who had terminated the employment relationship with a disabled person were also targeted. Furthermore, HR departments of large companies were contacted by the first author. After making an appointment, the interview participants received the study documents together with a consent form, which they signed before the interview. A total of 20 interviews were conducted with employers, see Table 1. All but two interviews took place at the workplace of the interviewee.

### Data collection

The interviews were conducted using a semi-structured interview guide. The following topics were covered in the interview guide, whereby the interview was initially very open and general and then increasingly structured and specific:

- Background information about the employer.
- Experience with affected employees (in-depth: support factors, barriers to sustainable work activity).
- Complementing and evaluating the themes derived from two literature reviews (enabling factors and barriers concerning person, work performance, work environment and environment in general) (21, 22).
- Further topics: Employer's responsibility for sustainable work activity, benefits for employer and employee, need for support.

The interviews, all conducted by the first author, were audio recorded and transcribed verbatim. During the interview, the interviewer also made notes in a grid comprising the following themes: Employee Characteristics, Employee Work Performance, Work Environment, and Environment.

### Data analysis

Sustainable employment is a central research topic in the work of the first and the last author. The researchers base their understanding of sustainable work on the definition of Finger and Fekete, which is based on previous group research and evidence in the literature (20, 35).

Data for employers of persons with SCI and persons with ABI were analyzed separately. The results of both analyses were compared and compiled in a second step. The coding grid was discussed and build up together by the first and last author during their familiarization with the data. The four basic areas, used in the interview grid, also formed the deductive coding grid for the analysis. Additional deductive codes were applied for the predefined themes of the interview guide “responsibility of employers,” “benefit for companies” and “need for support.” Within these areas, the themes that emerged in the interviews were coded inductively by the first author (a sociologist, experienced in qualitative research in the context of disability evaluation and SCI) (32, 36). Text passages were also coded by stage (reintegration or sustainable work activity) and assessment (facilitator or barrier). The first two interviews were coded independently by the first and last author (a sociologist, experienced in the topic of working with a disability and SCI). After five coded interviews, the first and last author jointly refined and specified the coding scheme. This step was repeated after all interviews were coded. Decisions and reflections were documented in the code book. In code memos, the first author described the analysis of the coded text passages and supplemented them with corresponding text passages. For the final step, the first author and the last author read all code memos and condensed the results. MaxQDA was used to support the analysis (37).

Data and inductive thematic saturation was reached for the research question on facilitators for sustained employment since no new themes arrived with coding of the last three interviews (38). However, saturation might not be reached for the topic of barriers to sustainable work activity, since we had difficulties to recruit enough employers where the person dropped out of work and therefore have overall less information on this aspect.

## Results

### Personal characteristics

From the employers' point of view, the personal characteristics of an employee with a disability that lead to a satisfying long-term work activity are, in particular, a suitable professional background, a younger age and the motivation to pursue a job. When employers perceive strong work motivation in an employee with an SCI or ABI, they are especially willing to support their return to work. An attractive professional qualification and experience profile can also lead to employers being more open to continue employment after the injury or to generally employ a person with a disability.

However, employers also indicated that they find the period of their employee's return to work after a spinal cord or brain injury challenging and often tiring, especially when the employee is struggling with the demands of his or her job. For example, an employee who is dissatisfied with adjusted work tasks or has had to accept a loss of responsibility may strain the working atmosphere, or a person who overestimates his or her abilities may make mistakes and thus harm the company. This phase of uncertainty is described by one of the interviewed employers:

[My employee with ABI] is extremely used to work and act independently and with high self-responsibility [due to her job position she had before the brain injury]. Of course, this is now a bit tricky. (ABI_10)

With regard to aging, employers point out that the older a person gets, the more negatively disability appears to affect job performance. Therefore, employers are also less willing to invest in older employees during the reintegration period. However, employers also point out that motivation, professional background and age are factors that are not only important for employees with a disability when it comes to sustainable work activity, but apply to all employees in general. In addition, employers expect their employees with a disability to have good self-assessment and, based on this, good self-management in dealing with the disability, meaning that the person is able to communicate his or her needs and generally handle all work-related demands with a minimum of assistance.

One employer described this characteristic as follows:

To achieve sustainability, the affected person should, whenever possible, become an expert in their own right. (SCI_ABI_01)

Employers emphasize the importance of the reintegration phase for sustainable employment in the long term. In this phase, the employer and the disabled person, both learn how to deal with the new situation. However, both employers and employees often lack knowledge and experience about how the disability affects work and how the affected person and the work environment should deal with it. Particularly in the case of people with ABI, employers emphasized their problems with the fact that major injury-related limitations are not directly visible. From the employers' point of view, it is therefore essential that the injured employees learn, with the support of professionals such as job coaches or vocational specialists in rehabilitation and work integration, to reflect on the impact of their disability on their work, to develop strategies for dealing with the impact of the disability and to identify their own needs, abilities and limitations. Employer expect that their employees, as experts in their own right, should ultimately know best how their work should be designed to meet their needs. Or as one employer put it:

It is not we employers who determine what work tasks a person can and cannot do. It's the employee who makes that decision. (SCI_03)

Employers also express how important it is for them that the person with a disability is able to describe his or her own situation and formulate work related needs:

There I had the feeling that it was not that easy for me to assess where [my employee with ABI] stands, but she supported me through her frequent feedback on how she experiences the situation. (ABI_07)

Such feedback helps employers to understand the situation of the person with a disability and to jointly and gradually find suitable tasks and forms of interaction during reintegration. Even in the case of a new job, employers appreciate it when the person with a disability proactively informs them about their own situation and points out possible solutions. In one case, according to the employer, the employment relationship failed precisely because a person with tetraplegia did not contribute actively to finding a solution for toileting at work. In addition, the person was not aware that the supervisor, who supported his employee with the catherization for some time, found the situation stressful. To assist in toileting the supervisor had to be available all the time, which interfered with his own need for flexibility:

The situation [i.e., the catheterizing] became too limiting and a problem for me. The real problem was that the affected person would not recognize that this was a challenge for me and showed no willingness to find a solution. (SCI_ABI_01)

Persons with a brain injury may have a limited capacity for reflection and self-awareness. This can manifest itself in the person overestimating their own performance at work. If the employer is not aware of the brain injured person's limited self-assessment ability and lacks professional support, this can lead to problematic work situations:

[The employee with ABI] wanted to do a good job [after returning to work]. He said he would manage this small construction project. But it was a disaster. He completely overestimated himself. It was the only time in 25 years that I have had a lawsuit over construction errors. (ABI_09)

Employers experience a discrepancy between their own performance assessment, that of the environment, and the self-assessment of the employee with disabilities as stressful.

A reduced capacity for reflection on the part of the employee, can also lead to disputes about workplace performance expectations and realistic performance delivery that challenge all parties involved. Such disputes can lead to profound conflicts and even result in the termination of the employment relationship. According to employers, professionals such as job coaches can play a mediating role here and point out possible solutions. However, if the person's performance falls short of the employer's expectations in the long run, even good support will not help.

### Work Performance

In the interviews, the employers repeatedly refer to the capacity of the person with a disability to perform their work and to create value for the company as the central factor for sustainable employment. Value creation is a central goal for any company, but it can manifest itself differently depending on the type of company: being in the black, producing a high-quality and usable output for a given amount of work, or simply balancing effort and return. For example, one of the employers interviewed said:

I recognize that the private sector should also create value, and therefore the employees should also create value or be able to contribute to the creation of value in the company. This is indeed the main focus of a company. In other words, I'm willing to employ someone in my company if I can see that they can support my company in some way with the strengths they have. And if I don't see that, then the person does not work for me. It's just not worth it. (SCI_ABI_01)

Or as one supervisor who works in a public company put it:

I think the limit would probably be where I would realize that my effort to support and develop the person is much higher than the output that I get back. (ABI_10)

Employers who employ a person with ABI or SCI ask themselves how the person's performance has changed as a result of the injury. The list of possible restrictions on work performance in the case of ABI is considerably longer than in the case of SCI according to the statements of the employers. Permanent fluctuations in performance due to concentration difficulties prove to be a particular challenge for employees with ABI and their supervisors, colleagues and co-workers, for example, when it comes to planning work processes. This is illustrated in the following example:

The instability is indeed a challenge. I cannot make a work schedule with this person for the next three weeks […] but we have ideas about what needs to be done and where she can contribute. But we actually have to adjust this on a daily basis. (ABI_07)

Another challenge for employers is to avoid overwork as much as possible, as this can have negative effects on the person's health and performance. Particularly for people with ABI, employers express that they were initially unsure to what extent they can put time pressure on the person. For example, one employer described:

We had to take away all tasks that involved short-term time pressure and just try to give him more long-term tasks. But there, too—at some point I needed a result there, too. This was a bit of a balancing act: ‘How much more consideration can I give?'. This is a challenge for me. (ABI_06)

The interviewees are also critical of longer health-related absences if they are unexpected and of indeterminate duration, although this was discussed more in the context of people with SCI. However, if longer absences, for example due to surgery, are planned in advance and agreed with all parties involved, this gives employers the necessary planning security. Employers cite the accumulation of sick days, for example also due to comorbidities such as mental illness, as a warning signal for a critical situation.

The employers are keen to achieve an as stable performance level as possible for the person with a disability during the reintegration phase, as this is a prerequisite for a “normal” employment contract. In this context, employers are quite willing to allow performance variations within an acceptable range as long as the overall performance is satisfactory. Or as this employer described it:

So, the one thing is [hesitates], as radical or dry as this may sound, but it is still a labor relationship for me. At the end of the day, it is based on ‘Work in exchange of a salary'. There is no issue with the employee having good and bad working phases. But at the end of the day the mutual expectations must be met. (ABI_04)

If performance expectations differ between the person with the disability and the supervisor, this leads to challenges. For example, during the reintegration phase, employers pointed out, that they tended to underestimate the impact of the disability and be overly optimistic about the long-term recovery of the disable person's ability to work.

It was especially challenging when at the same time, the employee indicated that he or she is perfectly capable of handling a higher workload and performing all previous work activities. In such cases, employers expressed their need for mediation by professional to guide their expectation management and if necessary for a “reality check” for the disabled person. Overall, employers see matching the resources and abilities of the person with a disability to the demands of the job as the basis for successful and sustainable work performance.

### Work environment

A suitable work environment is central to the sustainable employment of people with ABI or SCI. Accordingly, the employers interviewed addressed numerous aspects that can be assigned to the topic area of “work environment.” Of course, the employers are themselves part of the work environment. They can play a decisive role in shaping it and also see this as their responsibility:

Me as a direct supervisor—I think it is important that I create a work environment for this person that is appropriate for this person's capabilities. (ABI_06)

The work environment also includes, in particular, the work tasks that the person performs, the work organization (including workload, infrastructure and flexibility of work schedule), the work colleagues, the supervisor and the corporate culture.

#### Work tasks

For a sustainable work activity, from the employer's point of view, the intended work tasks must fit the person with a disability and his or her abilities so that the person can perform optimally. Employers emphasized that ideally, as soon as the injured worker returns to work, they try to put together a work package with tasks that make sense and are feasible for both the injured worker and the company. Depending on the limitations that a brain or spinal cord injury entails, this job crafting can be more or less challenging for the employer. For example, if a person is no longer able to perform their main job at a reception desk with a lot of customer contact due to the ABI, the work package is reduced and the work in the team must be reorganized, as the following example illustrates:

How can I find work packages that make sense both for the affected person and in the division of labor with the work colleagues? […] We actually had to remove her from customer contact, […] meaning she cannot work at the front desk anymore. […] This is a restriction for her, but also for getting the whole job done. (ABI_07)

The lack of suitable work packages for an employee is an obvious reason for the employer to terminate the employment relationship. From the employer's point of view, it is therefore important to regularly review and adjust the task package to ensure the person's long-term employment. It is also important that the person is able to grow in the task and maintain interest in the work:

If we talk about long-term employment, we simply have to offer them prospects that they can go into other areas. (ABI_05)

#### Work organization

In addition to the work tasks, the work organization must also be suitable for the person with a disability. From the employer's point of view, the following aspects are central to enable the person to remain in a job in the long term: an appropriate workload, usually on a part-time basis; a suitable infrastructure with accessible premises; and a high degree of flexibility in organizing working hours or taking the person's needs into account. These aspects are usually determined within the framework of vocational integration with the support of professionals.

Employers who can *offer part-time positions* with a low workload of 20–40 percent provide special opportunities for those affected who are looking for a new job. This might more likely be the case with state-owned companies than with private companies. However, it should not be neglected that even with smaller workloads there are costs for the infrastructure:

The person needs a desk, a computer, an infrastructure. We offer that, even if the person only has a 15 percent workload. (ABI_07)

The funding of *infrastructure adaptations* by the Swiss disability insurance (IV) allows employers to adapt their premises to meet the needs of disabled employees, with adaptations being more comprehensive for persons with SCI than with ABI. However, these adaptations are often delayed and minimally implemented by the IV, especially when it is uncertain how long a company will remain in a location or how long the person with a disability will be with the company. Therefore, one employer is calling for more generosity in infrastructure adaptations that work not only for the individual case, but also for new employees with a disability:

If one takes into account the added value—that the affected person is integrated, has an income and so on, I sometimes found it a bit petty and tedious when they [the disability insurance authorities] said ‘It's sufficient if [the person with a disability] can enter from the back door'. […] So, they are reluctant. So, they save money with regard to the particular case at hand and do not realize that it might be good if they were a bit more generous with the employer. Because the employer might then later have the means to reintegrate yet another person [with a disability]. (SCI_ABI_03)

Sometimes employers simply do not know that the IV provides funding for infrastructural adaptations, or they do not know how to apply for funding when they employ a person with a disability.

In addition to a reduced workload and suitable infrastructure, employers try to allow *flexible working hours*, breaks as needed and home office so that the person with a disability can respond quickly to problems caused by the ABI or SCI, for example by leaving the workplace earlier if needed. Organizing and planning work is often perceived as a challenge by employers and involves a lot of effort, as describe by the following employer:

That is our issue at the moment—How do we organize work so that [the employee with ABI] is not overworked, does not get stressed out? Precisely also because she's not next to me all day, I don't see her often. She works a lot overtime. That's not good [laughs], I would say. Luckily, she keeps a record of her hours, so I can see how much overtime she works. But to estimate how much work I can give her so she can deal with it—that is incredibly difficult. (ABI_10)

#### Work colleagues and supervisor

The knowledge and awareness of the supervisor and work colleagues about the specific effects of the ABI or SCI on the functioning of the person are central to establishing structures and processes in which both the affected employee and the entire work environment feel comfortable. Inexperienced employers particularly appreciate the support of integration professionals, such as job coaches, who impart knowledge and raise awareness or support the affected person in raising awareness among persons in his or her work environment. From the employers' perspective, it is crucial to maintain this awareness to ensure long-term success in the workplace:

[The employee with SCI] may approach his supervisors at any time if something bothers him. They have been working together for four years. Also, the new supervisor he has—they have worked together before. It's not someone external who does not know the situation. (SCI_06)

The phase in which an affected person gradually returns to work can tie up considerable resources of the employer and work colleagues. The interviews revealed that both employers and work colleagues are very committed at the beginning of the integration process, provide help or step in when needed, and do not perceive this as a burden. There is a common goal, that is seen as a “project” to which everyone contributes. However, as the project progresses and the injured person's performance does not meet expectations, there is a risk that the willingness to support will wane:

The work colleagues step in, they bridge the gap during the absence of the [person with ABI]. When he returns to work, everything seems to be ‘on green', meaning everything is going well. And now it is a bit of a moment where the affected person himself cannot quite cope with the situation. We now realized that it is actually difficult for his work colleagues not to start seeing it as a burden. (ABI_04)

The employers interviewed see their own role as supervisors in a long-term employment relationship primarily in making time resources available to the person with a disability, whether through a sympathetic ear, more frequent performance feedback or additional support in dealing with the Swiss disability insurance and organizing further training. Or as the following employer describes it:

It's the responsibility of the supervisor to take the time to support the person to stay at work and keep up with the changes in the company like Windows 365 and digital communication. That is challenging at the moment. It's important that one does not simply think ‘Ok they are now just a bit limited in this respect or have a bit of trouble', but one has to say ‘This person is an equal employee, just like any other'. It just takes a bit more attention, time, guidance and inquiries. (ABI_10)

#### Corporate culture

Both the way employers and work colleagues deal with employees with ABI or SCI and the possibilities for organizing work according to individual needs are anchored in the specific corporate culture. The interviewees speak of an inclusive corporate culture when the company is interested in the general wellbeing of the employees, offers support in case of problems and is open toward hiring persons with a disability:

Our cooperate culture is characterized by generous services and benefits for our employees […]. [The inclusive cooperate culture] is exemplified by our top management, therefore it is also established within the whole company. (ABI_04)

The specific corporate culture depends on the type of company. Employers of state-owned companies, for example, see a higher responsibility for their company in the integration of persons with a disability. And larger companies usually have their own HR-departments, strategies and instruments, such as corporate health management, which provide resources for the integration and support of persons with a disability. However, these departments tend to focus on the reintegration of injured company employees rather than on the recruitment of people with a disability per se.

For private companies, financial health plays a crucial role. If the financial situation is good, the willingness to provide resources for the integration of a person with a disability increase. From the employer's point of view, a corporate culture that is described as ‘family-like' is also beneficial, as this is associated with greater closeness between management and employees and an increased interest in the wellbeing of the individual. Companies that are themselves active in the field of disability and work integration see their responsibility in employing people with disabilities in order to act as role models and gain experience.

The communication culture in the company is also very important. Employers emphasize the importance of an open, transparent communication that involves all employees, especially when it comes to addressing and clarifying problems related to the job performance of the person with a disability:

There certainly needs to be someone responsible for handling the co-workers [of the person with a disability], who can act as a mediator and explain things. I believe that it often leads to problems among co-workers when someone does not perform as expected. [The co-workers] then come right out and say ‘He is privileged and we have to do the hard work'. In such cases there is a need for explanation. (ABI_08)

However, as far as transparency is concerned, the interviews reveal different views. While most are in favor of as much transparency as possible (for example the impact of the disability on work performance should be discussed), one employer (ABI_09) fears that too much transparency will put the focus on the limitations rather than the person's abilities.

If a company already has an open and inclusive corporate and communication culture, this facilitates the integration of people with a disability, especially when it comes to new applicants. Conversely, the corporate culture can be further developed and strengthened through positive experiences with a disabled employee, according to the following employer:

In the case [of our employee with SCI] we also succeeded in shaping the cooperate culture a bit further, in the sense of ‘What would you wish for if you were in this situation'? and that's how we also act as a company, or at least we try to act that way. (SCI_06)

On the other hand, the inclusive values of a company can also be challenged due to a negative experience, as was the case with the following employer, for whom the reintegration of an employee with SCI and ABI had to be stopped:

I would say we have been doing the social thing [i.e., the reintegration of persons with a disability] quite intensively for some time. But we also got a lot of flak. And that has moved us to the point that we have said: ‘It's definitely not our job to help everyone'. We are still doing it, it's not like there was a 180-degree turnaround. We simply realized ‘Wait a minute, you are going to get hit on the head at the end, or somehow at the end of the year the numbers do not add up at all, but that can't be it'. We look much more closely now, where before we were perhaps a bit naïve. (SCI_ABI_02)

A change in management and an accompanying change in corporate culture, for example toward more focus on efficiency, can be a risk factor, as this supervisor describes:

The new managing director says [about the employee with ABI], ‘His performance is in the red, this is crap, how do we deal with it?'. So, the [employee with ABI] faces a direct threat from the new managing director. (ABI_09)

According to the employers, the creation of a new “normality” is an important factor for a successful long-term employment of a disabled person. This means that there is an established way of dealing with the disability on the part of both the work environment and the person with a disability, which no longer needs to be discussed in everyday work. In the organization of work, the needs of the person with a disability are automatically taken into account in the daily planning. This also applies, for example, to the planning of events such as a company party. One employer describes the striving for normality in dealing with the person with a disability as follows:

I think that in the long run, it really helps to see each other as normal co-workers, so as to establish normality. So that dealing with [the disability] is something normal. (ABI_04)

Changes in the work environment such as new colleagues or changes in work tasks and procedures can disrupt this “normalcy,” especially for persons with ABI. For the employers interviewed, these changes increase the risk for drop-out, especially if there is a lack of sensitivity or effort to communicate and address the needs of the disabled employee in the new situation.

### Additional environmental factors

Employers mentioned other environmental factors outside the work environment that can have a positive or negative impact on the work situation and job performance of the person with a disability. For example, when a person returns to work after a brain or spinal cord injury, *support from medical and vocational professionals* during rehabilitation and vocational reintegration is essential from the employer's perspective. At the beginning, employers and affected persons often lack the knowledge to design the work environment according to the person's needs. Employers therefore appreciate a specialist as a contact person who facilitates a regular exchange with all parties involved and raises awareness among superiors and work colleagues. The specialist also mediates in the event of problems and disagreements, supports the self-efficacy of the person with a disability and helps with insurance issues. Employers who did not receive professional support during the integration process, for example in the form of job coaching, describe the situation as overtaxing and, in retrospect, perceive themselves as “naïve.” Employers also experience it as negative when there is no competent contact person and the coordination of the integration is unstructured, as was the case with the following employer:

With the first case [of an employee with ABI] there was now structure from the hospital. […] We also had no contact person at the hospital. (ABI_06)

Some employers point out that they appreciate having access to ABI and SCI specialists even after successful reintegration of a disabled employee if new problems arise.

Regarding the two major Swiss *disability insurers*, the Swiss Accident Insurance (Suva) and the Swiss Disability Insurance (IV), the financially better-off Suva is perceived by employers as more supportive of occupational integration than the IV, as this employer's statement shows:

The Suva was very straightforward. They were so happy that we did not make a welfare case [out of the employee with ABI]. With the IV it was more bureaucratic. With the IV I did not get the feeling of having a case manager who really cares about the case, instead it's somehow just a stupid numbers game. (ABI_09)

Nevertheless, with the IV being legally mainly responsible for financing vocational integration measures and workplace adjustments in Switzerland, the employers appreciate its financial support for infrastructural adjustments to improve accessibility and in the form of daily allowances for employees undergoing retraining. While some employers have had good experiences with individual case managers, cooperation with the IV is generally perceived as rather difficult, as the following example shows:

To come back to the IV topic [laughs]—I find the whole thing a relatively cumbersome apparatus. If you have someone who works there top motivated […], then it's super. They tell you ‘Call us if there is anything'. In this case [with our employee with ABI] the support [from the IV] is great. But then there are others that you just cannot reach by phone. And also, the early detection tool they have [Früherfassung]. It takes so long until you get something tangible out of it. (ABI_02)

Employers also see a problem with the bad image of the IV, as the example of the following employer shows:

Do employers want to have the IV in the house? […] The disability insurance does not have such a great name. It is still something stigmatizing. (ABI_08)

Employers do not consider the IV as the first point of contact when problems related to an employee's disability arise at the workplace, even though the IV would be officially responsible in such cases and would also have the appropriate means to intervene at an early stage. Employers are unsure whether they would receive competent, direct and sympathetic support in a timely manner. From the employers' point of view, the IV procedure related to employing a disabled person is associated with a high administrative burden, is very technical and is too strongly geared toward optimizing the financial situation of the IV. In particular, the determination of a partial or full pensions and subsequent pension revisions, which take place approximately every 5 years, are associated with uncertainty for the employer and the person with a disability, absorb the person with a disability strongly during this time and cause additional stress, which leads to lower work performance.

Besides the specialist support and issues related to the disability insurance schemes, employers mention the *family situation* of the person with a disability as a decisive factor that can influence job performance. The family can have a relieving or burdening effect on the affected person. Employers are sometimes well informed about the family situation, as there is an increased exchange between employers and employees due to the disability. More free time thanks to relief in the private sphere is beneficial, but family problems and disputes can lead to a reduction in performance:

For [the employee with ABI] the private environment is a very important point. If that were better, he might be able to work even more than 50 percent. (ABI_03)

Employers also perceive that *financial obligations* can also have a negative impact on job performance by putting pressure on the person with a disability to work a high workload. An adequate IV pension therefore makes it easier for the person with a disability to decide not to exceed a feasible workload.

Challenges due to a competitive and performance-oriented *labor market* and general reservations of employers toward jobseekers with a disability are perceived by the interviewees mainly with other employers. In contrast, their own experiences with ABI and SCI workers have led them to overcome their own reservations for the most part.

### Mutual benefits

For the employers, there is a win-win situation in the long run when they receive a work benefit and the person with a disability has an opportunity to pursue employment as the following employer says:

The benefit is certainly the work output that I get. But it is also the positive feeling that I could give a chance to a person, that I could integrate that person and give her back the place in society she wants. (ABI_08)

Employers also see a benefit in the affected person's loyalty and gratitude toward the employer, which they believe can translate into higher motivation and job performance:

The willingness to perform and the ability to perform are the same, if not higher, for persons with a disability [as compared to persons without a disability]. (SCI_ABI_03)

## Discussion

The aim of the study was to show which factors, from the employer's point of view, are central to the sustainable employment of people with ABI or SCI in Switzerland.

For the employer, the final focus is on the performance of the person who contributes to value creation in the company. Discrepancies between the employee and the employer in terms of performance expectations and evaluation, as well as prolonged absences from work, are correspondingly central risk factors for a drop-out. If the affected person has good self-management of the disability and good communication skills, this strongly supports the employer, as he/she lacks knowledge about the effects of the disability, especially at the beginning. If the person with a disability lacks communication skills, support, for example with job coaching, helps to mediate between employer and employee. Support from specialists during the vocational integration process was particularly important for employers who had no previous experience with employees with a disability. The importance of communication skills and self-efficacy in relation to their disability is also highlighted in the literature when discussing sustainable employment and job change among persons with a disability (39).

Employers in this study see themselves as responsible for offering affected persons an optimal working environment. This includes, for example, suitable work tasks and opportunities for further development, an open and sensitive work team and flexible work organization. Equally central are the person-job match, the availability of resources for addressing the specific needs of the person with a disability and an inclusive corporate culture in which the employment of and interaction with a disabled person is a matter of course. These findings are consistent with other research studies, including those on employer perceptions (25, 27).

Differences between people with ABI and SCI are evident in terms of the direct impact of the disability on work performance. For people with SCI, physical limitations are the primary concern. For people with ABI, the possible consequences are more varied and can be physical, but often also cognitive. The vocational integration of people with ABI and cognitive impairments is experienced as more difficult and protracted by employers who have experience with both people with ABI and people with SCI. This is related to the fact that the consequences of the ABI are less well understood by both the person with a disability and all the other parties involved. Brain injury further complicates this process when self-reflection and self-awareness are affected negatively. The lack of visibility of the consequences of ABI and in some cases SCI, for example when incontinence is an issue, means that vocational integration must be accompanied by specialists. In the course of this study, it has become clear that support for people with SCI in vocational integration and later working life is much better established and covered by a wider range of services than for people with ABI in Switzerland.

The aspects raised by the employers interviewed in connection with sustainable employment largely coincide with the findings of existing international studies that look at the period of vocational rehabilitation or working with ABI or SCI in general (21, 22). In addition, these studies also found that many aspects that our study identified as important for a sustainable work ability are initiated and established during the vocational reintegration phase. Employers stressed that raising awareness of the work environment and the availability of flexible work options are paramount for smooth, sustainable collaboration with people with ABI, while performance fluctuations and challenges related to the consequences of ABI are problematic in the long run (22, 40). For people with SCI, the importance of self and health management and adaptations in the work environment, as well as support from work colleagues, is emphasized. Barriers to sustainable work ability relate to difficulties in work organization and social discrimination (21).

Specific, though not surprising, the interviewees focused strongly on performance as a critical factor to sustained employment and the challenges associated with discrepancies in performance expectations between employers and employees, what was also pointed out by Gilbride et al. (26). The interviews illustrated that particularly certain changes (for example new employees, new processes for people with ABI or new premises for people with SCI) are threatening for sustainable employment. Such changes may lead to drop-out if not identified and resolved in time. Employers' lack of knowledge and the invisibility of disability-related functional limitations, particularly for employees with ABI, have been identified as important reasons for unsustainable work in a previous study (41). Similarly, Bonaccio et al. (28) address some aspects that were also raised by our interviewees, such as the lack of knowledge about the consequences of disability, fear of additional work, for example in connection with infrastructure adjustments, and the importance of raising employees' awareness. However, Bonaccio et al. also point to additional barriers that prevent people with a disability from accessing jobs. These include, for example, a discriminatory recruitment process or incorrect assumptions about the qualifications of people with a disability, what was not addressed by the employers in this study. As a result, the resources of persons with a disability lie idle due to employers' fears. The employers taking part in our study pointed out that positive experiences with disabled employees helped them to leave their prejudices behind and become more open to recruiting people with a disability, especially when supported by an organization that focuses on the work integration of persons with a disability.

Overall, it is important to highlight that the employers who participated in the present study tend to represent largely positive examples of work integration and inclusion of persons with a disability. Most of them either had a personal relation to ABI or SCI, were accompanied by professionals during the vocational reintegration process or are part of a network of organizations dealing with the issue of work and disability. These characteristics of employees are typical of employers who are willing to hire and support workers with a disability (23, 25).

### Limitations

No new information emerged in the last interviews, meaning that data saturation on our research question was reached. Nevertheless, because it was difficult to identify and recruit employers who had negative experiences with disabled workers, the saturation on the topic of barriers to sustainable work activity can be questioned. The interviewees also showed a strong tendency to return again and again to the reintegration phase, because it was in this phase that they had dealt most with the disability and the consequences for their work activity, and it was there that a great deal had been initiated that would become central to sustainable work activity. However, there is evidence that once a stable work situation has been reached, the starting point of gainful employment (return to the old employer, to a new employer or entering the labor market for the first time) fades into the background (42). Therefore, we only documented whether the employer hired a person with a disability or continued to employ an employee after the injury. Although we carefully separated the analysis of data from employers of people with SCI and ABI, there may remain a risk of bias in the results when combining the experiences of employers of people with ABI and SCI.

### Implication for future research

Longitudinal interview studies dealing with the work biography of persons with ABI or SCI from the employer's perspective could provide additional information on critical occurrences, there solutions and possible external support needs. In addition knowledge on transitions between work activities, could inform recruitment strategies. For example, this study only marginally addresses the issue of job change when it comes to a person's recruitment and termination of employment. In-depth studies of diversity and health management policies in larger companies could also shed light on how they are implemented for persons with a disability and to what extent they actually have a positive effect on their employment, as research has shown a discrepancy between attitudes and behaviors in hiring persons with a disability (25). A still better understanding of the employer's perspective is of central importance for the further promotion of sustainable employment for persons with ABI or SCI.

## Conclusion

This study identifies critical aspects that contribute to sustainable work activity for people with ABI or SCI, such as needs-based work environment design and coordinated vocational rehabilitation to prepare employers and employees for a long-term sustainable work situation. From the employer's perspective, people with a disability should be able to communicate their work-related needs and take care of their own health in the long term so that any problems that arise can be addressed as early as possible. Supportive is also a continuous sensitization of the environment. In addition, the expansion of low-threshold health-specific support services for long-term problems was found to be of great importance to employers in Switzerland to help them avoid work loss in the long term and to promote sustainable employment of people with ABI or SCI.

## Data availability statement

The raw data supporting the conclusions of this article will be made available by the authors, without undue reservation.

## Ethics statement

The studies involving human participants were reviewed and approved by Ethical Committee of North-West and Central Switzerland (EKNZ, study reference 2018-01317). The patients/participants provided their written informed consent to participate in this study.

## Author contributions

BS, BT, and MF were responsible for designing the conceptual framework of the study. BS and SS recruited participants. BS conducted and transcribed the interviews. BS coded the interviews and together with BT analyzed the data. Results were discussed with MF, KK, and SS. BS, MF, and BT prepared the paper and all authors gave feedback on the paper. SS provided valuable input from vocational rehabilitation practice. All authors contributed to the article and approved the submitted version.

## Funding

This work was supported by the Swiss National Science Foundation under Grant No. 10531C_173322/1.

## Conflict of interest

The authors declare that the research was conducted in the absence of any commercial or financial relationships that could be construed as a potential conflict of interest.

## Publisher's note

All claims expressed in this article are solely those of the authors and do not necessarily represent those of their affiliated organizations, or those of the publisher, the editors and the reviewers. Any product that may be evaluated in this article, or claim that may be made by its manufacturer, is not guaranteed or endorsed by the publisher.
